# Association of Blood Mercury Level with the Risk of Depression According to Fish Intake Level in the General Korean Population: Findings from the Korean National Health and Nutrition Examination Survey (KNHANES) 2008–2013

**DOI:** 10.3390/nu12010189

**Published:** 2020-01-09

**Authors:** Kyung Won Kim, Sundara Raj Sreeja, Minji Kwon, Ye Lee Yu, Mi Kyung Kim

**Affiliations:** 1 Department of Food and Nutrition, Seoul Women’s University, 621, Hwarang-ro, Nowon-gu, Seoul 01797, Korea; kwkim@swu.ac.kr; 2 Division of Cancer Epidemiology and Prevention, National Cancer Center, 323, Ilsan-ro, Ilsandong-gu, Goyang-si, Gyeonggi-do 10408, Korea; ssreejaraj@gmail.com (S.R.S.); yelee216@ncc.re.kr (Y.L.Y.)

**Keywords:** blood mercury, depression, fish intake, Korean National Health and Nutrition Examination Survey

## Abstract

Mercury is a cumulative neurotoxic agent, exposure to high levels of which may increase the risk of psychiatric symptoms. The purpose of this study was to examine the associations between blood mercury and depression risk in Korean adults. We analyzed the Korean National Health and Nutrition Examination Survey (KNHANES) with 11,754 participants (male: 5834 female: 5920) aged ≥19 years from 2008 to 2013. The associations of blood mercury with risk of depression were estimated using multivariate logistic regression after adjustment for potential confounders. We found a significantly increased risk of depression in the highest quintile for blood mercury (multivariate OR = 2.05; 95% CI = 1.20–3.48; *p* trend = 0.03) among female, but not male. A stratification analysis by fish intake showed that the association between depression and blood mercury was strengthened (OR = 4.00; 95% CI = 1.51–10.6; *p* trend = 0.015) among females with the lowest tertile of fish intake. The results of this study suggest that higher levels of blood mercury, especially in cases of lower fish intake, are positively associated with the risk of depression in Korean women.

## 1. Introduction

Mercury is a ubiquitous neurotoxin that exists in elemental, organic, and inorganic forms in nature [1,2]. The mechanism involved in methylmercury toxicity are inhibition of protein synthesis, alteration of protein phosphorylation, and microtubule disruption, resulting in increased intracellular Ca2+ concentration that leads to alteration of neurotransmitter function and neuronal death [3]. Humans incur the highest mercury levels from consumption of seafood because, while methylmercury is accumulated from plankton to humans at high absorption rates, mercury’s excretion rate from the human body is very low and its retention time is long [1,4]. Exposure to mercury, in its effect on the nervous system, increases the risk of psychiatric symptoms [3]. An epidemic outbreak of methylmercury poisoning was first reported in 1956 among residents around Minimata Bay in Japan, the symptoms being severe behavioral dysfunction, deterioration of cognitive function, changes in mood levels, and paralysis, resulting, eventually, in some cases, in death [5]. Moreover, among Iraq residents in 1972, consumption of methylmercury-treated wheat flour led to neurological deficits, symptoms of depression and anxiety, loss of co-ordination and hearing, as well as slurred speech and blindness [6].

Fish is one of the major food sources of polyunsaturated fatty acids (PUFA) such as omega-3 fatty acids including eicosapentaenoic acid (EPA) and docosahexaenoic acid (DHA) [7,8,9]. In humans, EPA and DHA are synthesized from alpha-linolenic acid (ALA), which in turn is obtained from diets [10]. According to the American Cancer Society (ACS) and the American Heart Association (AHA), consumption of fish at least two times a week is recommended [11]. The omega-3 fatty acid is an essential nutrient; it has been suggested, in fact, that sufficient consumption of fish has a protective effect against cardiovascular disease (CVD) and mental illness such as depression [7]. Omega-3 fatty acid deficiency leads to impairment of serotoninergic and dopaminergic neurotransmitters and, thereby, altered inflammatory status of neurological functions [12,13]. According to the Birth Cohort Study in Finland, inappropriate PUFA consumption incurs a higher risk of depression among women [14]. Methylmercury, a principle type of organic mercury, accumulates in the human body through fish intake, and more than 99% of blood mercury level is known to be due to fish consumption [15,16]. Several studies have revealed that higher intake of fish is significantly associated with increased risk of mercury exposure [1,17,18,19].

Studies of blood mercury levels have shown both significant and inverse associations of mercury with depressive risk and symptoms [20,21]. In Iraq, total blood mercury level was reported to be higher in depressed patients with symptoms of depressive behavior, lack of interest, and deficient concentration [20]. Moreover, study participants from the Minimata area of Japan, who had been highly exposed to methylmercury, showed a higher prevalence of depressive and psychiatric symptoms than did participants from other areas [21]. By contrast, among older adults in the general American population, lower risk of depression was associated with higher levels of blood mercury [2]. Overall, the findings from epidemiologic studies on possible links between mercury and depression risk are inconsistent and mixed.

The aim of the present study was to examine the association between mercury and depression risk and to determine whether it differs by fish intake level and gender using the Korean National Health and Nutrition Examination Survey (KNHANES) 2008–2013.

## 2. Materials and Methods

### 2.1. Study Design and Population

The KNHANES is a national cross-sectional survey conducted by the Korea Centers for Disease Control and Prevention (KCDC) to evaluate the health and nutritional status of the Korean population since 1998 [22]. It monitors the trends in health risk factors and the prevalences of major chronic diseases, and also provides data for the development and evaluation of health policies and programs. The target population is the non-institutionalized civilian Korean population. In order to obtain representative samples, the sampling plan follows a multi-stage clustered probability design that includes cohorts aged ≥1 year. The KNHANES consists of a health interview, a health examination, and a nutrition survey for collection of data on anthropometric, biochemical and clinical profiles and dietary intakes. Further details on the design, methods, and survey contents are available in the literature [22]. The KCDC has published the Korea Health Statistics every year, and the data are publicly available on the KNHANES website (http://knhanes.cdc.go.kr).

**Figure 1:**
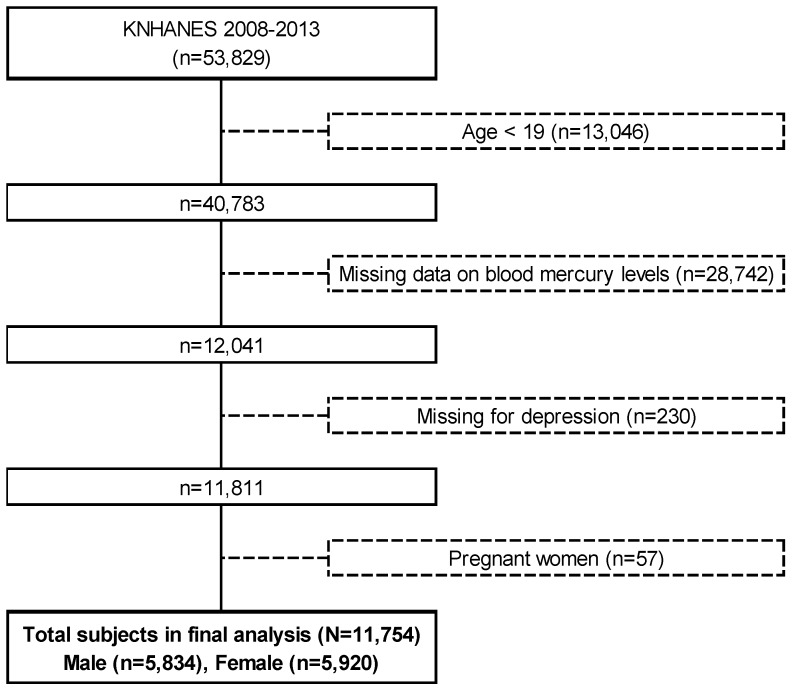
Flow diagram of selection of participants for inclusion in the present study (KNHANES 2008–2013).

This study was based on the KNHANES 2008–2013 including KNHANES IV (2008–2009), KNHANES V (2010–2012), and KNHANES VI (2013). A total of 53,829 individuals took part in KNHANES 2008–2013. We excluded subjects younger than 19 years of age (*n* = 13,046), those who failed to report blood mercury levels (*n* = 28,742), those suffering from depression (*n* = 230), and pregnant women (*n* = 57). The present study evaluated 11,754 adults aged ≥19 years for whom there were complete responses to the health interview, health examination, and nutrition survey regarding blood mercury levels, symptoms of depression, frequency of fish intake in the last year and other covariates such as age, gender, tobacco smoking, alcohol consumption, household income, physical activity, and energy intake (Figure 1).

This KNHANES study was approved by the Institutional Review Board of the KCDC (2008–2013) (IRB No. 2008-04EXP-01-C, 2009-01CON-03-2C, 2010-02CON-21-C, 2011-02CON-06-C, 2012-01EXP-01-2C, 2013-07CON-03-4C) and was conducted in accordance with the Declaration of Helsinki. It was exempted from the requirement of participants’ written informed consent because the dataset did not include any identifiable personal information and consent had already been given to KNHANES. All information collected is strictly protected by laws.

### 2.2. Depression Assessment

The variables for depression were collected in the health interview by trained interviewers. Information on individual mental health was obtained using the structured questionnaire Patient Health Questionnaire9 (PHQ-9), which was adjusted according to the standard definitions [23,24]. The presence of depression was determined by the question; “Have you ever been diagnosed with depression confirmed by a physician”?

### 2.3. Measurements

#### 2.3.1. Determination of Mercury Levels in Blood

Blood samples were collected at a mobile examination center. The participants’ blood samples were drawn into sodium heparin tubes after an overnight fast. Blood mercury levels were assessed by the gold-amalgam collection method using the Direct Mercury Analyzer 80 (DMZ-80, Milestone, Bergamo, Italy). All of the blood mercury analyses were performed by the Neodin Medical Institute, a laboratory certified by the Korean Ministry of Health and Welfare. The Limit of Detection (LOD) for blood mercury was 0.158 µg/L. No sample exhibited values below the LOD. The inter-assay co-efficient of variation for the mercury assay ranged from 0.47 to 6.08%.

#### 2.3.2. Dietary Fish Intake Assessment

Fish intake was assessed with a qualitative food frequency questionnaire (FFQ) that had been completed by recording the details of fish consumption during the year prior to the nutrition survey [25]. The FFQ consisted of 63 commonly consumed food items in Korea with frequencies of intake. The frequencies of fish intake were classified into ten categories: almost never, six to eleven times a year, once a month, two or three times a month, once a week, two or three times a week, four to six times a week, once a day, two times a day, and three times a day. “Fish” included white fish (croaker, pollack, hairtail), fatty fish (mackerel, tuna, anchovy), other fish (fish cake, squid, salted seafood), and shellfish (clam).

#### 2.3.3. Other Covariates

A wide range of covariates were assessed in this analysis. The anthropometry on height and weight was conducted in the health examination, and the questionnaire related to socio-demographic characteristics was administered in the health interview. The socio-demographic characteristics included were age, gender, body mass index (BMI), household income, marital status, alcohol consumption, tobacco-smoking habits, and physical activity. Age was used as a continuous variable. Gender was divided into male and female. Household monthly income was categorized into four groups, including low (<750,000 won), low-intermediate (750,000–1,500,000 won), upper-intermediate (1,500,000–2,463,000 won), and high (>2,463,000 won). Marital status included married and single. Alcohol intake was categorized into four groups, including non-drinker, ≤1 time/month, ≥2 times/month, and heavy drinker. Smoking status was classified into three groups: non-smoker, past-smoker, and current smoker. Individuals with BMI < 18.5, 18.5 to <23.0, 23.0 to <25.0, and ≥25 kg/m^2^ were defined as underweight, normal, overweight, and obese, respectively. The International Physical Activity Questionnaire measures moderate- and vigorous-intensity physical activity, and has tested reliability and validity with objective measurement tools [26]. High physical activity was defined as participating in moderate-intensity physical activity for at least 2 h and 30 min, vigorous-intensity physical activity for more than 1 h and 15 min, or a combination thereof (1 min of high-intensity activity is defined as 2 min of moderate-intensity activity) over a period of 1 week. The physical activity of the subjects was quantified in terms of metabolic equivalent of task minutes per week (MET-min/week), which was calculated using the scoring protocol of the Korean version of the International Physical Activity Questionnaire short form [27].

### 2.4. Statistical Analysis

Survey data from KNHANES 2008–2013 was combined to form a single dataset. Blood mercury was divided into quintiles for analysis. The baseline characteristics of the study subjects were presented as mean and standard error for continuous variables and percentages for categorical variables. The ANOVA and chi-square tests were used to analyze the differences in the distributions of the continuous and categorical variables, respectively. We divided the subjects into quintiles of blood mercury in each gender. We performed the analysis of shape of association between mercury and depression using restricted cubic spline [28]. It showed a linear association, and then odds ratios (ORs) and 95% confidence intervals (CIs) were estimated using survey-weighted multivariate logistic regression analysis for the association between depression and blood mercury according to the following covariates: model 1 was adjusted for age, physical activity, and energy intake as continuous types, and for gender, household income, drinking, and smoking as categorical types; model 2 was adjusted for the covariates of model 1 including fatty fish intake as a continuous type; model 3 was adjusted for the covariates of model 1 including total fish intake as a continuous type. *p*-values for trends were calculated according to quintiles of blood mercury levels. Survey-weighted logistic regression was computed to analyze the association between depression and blood mercury based on fish intake, according to the following covariates. Total fish intake was divided into tertiles based on gender; the age-adjusted model was adjusted for age as a continuous variable; the multivariate-adjusted model was adjusted for physical activity and energy intake as continuous variables and for smoking status, drinking status, and household income as categorical variables in addition to the covariates in the age-adjusted model. We used SAS SURVEYREG and SAS SURVEYLOGISTIC for the survey-weighted logistic regression analysis. All of the statistical analyses were performed using Statistical Analysis System software 9.4 (SAS Institute Inc., Cary, NC, USA). The reported *p*-values were 2-tailed; values less than 0.05 were considered to be statistically significant.

## 3. Results

The distributions of the general characteristics of the study subjects by quintile of blood mercury are shown in Table 1. Blood mercury level was significantly related to age (*p* < 0.0001), BMI (*p* < 0.0001), marital status (*p* < 0.0001), smoking (*p* = 0.02), drinking (*p* < 0.0001), household income (*p* < 0.0001), physical activity (*p* = 0.03), and intake of fish, white fish, fatty fish, and other seafood (*p* < 0.0001). Subjects in the highest quintile of blood mercury relative to those in the lowest quintile included adults who were older, female, more likely to be obese, more likely to be married, heavier drinkers, and who had higher household income. With respect to fish intake, the frequency of fish consumption increased as blood mercury levels increased. Individuals in the highest quintiles of blood mercury consumed white fish, fatty fish, and other seafood more frequently than did those in the lowest quintile.

The multivariate logistic regression results of the relationship between depression and blood mercury are presented in Table 2. For the total subjects, the participants did not show any significant association between blood mercury and depression, despite the multivariate analysis (model 1) controlling for demographic and lifestyle variables. However, a statistically significant association between depression and blood mercury was noted after adding fatty fish intake (OR = 1.77; 95% CI = 1.12–2.78) (*p*-trend = 0.02) and fish intake (OR = 1.76; 95% CI = 1.12–2.76) (*p*-trend = 0.03) to the multivariate adjustment of demographic and lifestyle variables. When the multivariate logistic regression analysis was performed by gender, the association between depression and blood mercury differed. Females showed a significant association between depression and blood mercury in model 2 (OR = 2.07; 95% CI = 1.22–3.51) (*p*-trend = 0.03) and in model 3 (OR = 2.05; 95% CI = 1.20–3.48) (*p*-trend = 0.03). By contrast, among males, blood mercury was not significantly related to depression in any of the models examined.

Table 3 provides the odds ratios and 95% confidence intervals for the association between depression and blood mercury as stratified by gender and fish intake. Total fish intake was divided into tertiles by gender. In females, the association between depression and blood mercury differed by a tertile of fish intake. In the lowest tertile of fish intake among females, the association between depression and blood mercury was strengthened in the multivariate-adjusted model (OR = 4.00; 95% CI = 1.51–10.6) (*p*-trend = 0.01). However, the odds ratios and 95% confidence intervals were not significantly increased as the quintiles of blood mercury increased among females with greater fish intake. However, in males, depression was not associated with blood mercury in either the age-adjusted or multivariate-adjusted model by tertile of fish intake.

## 4. Discussion

In this study, we analyzed the relationship between blood mercury levels by fish consumption and depressive symptoms using KNHANES 2008–2013. Higher mercury level was associated with increased risk of depression in Korean adults; however, the association varied by fish intake and gender. After multivariate adjustment, females showed a positive association between blood mercury and depressive symptoms. A significant predominant association was observed between blood mercury and depression among female participants with low fish intake.

Several studies have reported that mercury, which is generated by human activity and natural environmental change, causes several adverse health effects in humans [29,30,31,32,33]. Methyl mercury is absorbed in the gut and dispersed to the whole body by mixing with red blood cells, and is excreted from the body via urine and feces. Both organic and inorganic forms of mercury, which can be transported through the blood–brain barrier and from the placenta to the fetus, induce neurotoxic symptoms [18]. Higher exposure to the inorganic form of mercury, especially methylmercury, causes neuropsychiatric symptoms by eliciting oxidative stress in the central nervous system [29]. Apart from this, mercury also induces symptoms of gingivitis, stomatitis, dermatitis, ischemic stroke, and dementia in affected individuals [30,31,32]. Additionally, it leads to gastrointestinal toxicity, nephrotoxicity, risk of atherosclerosis and cardiovascular diseases, and depression [18,33,34].

Dietary intake, environmental factors, and conditions are believed to increase the blood mercury levels in human beings. Mercury level in the blood of the general population are derived mainly from the dietary intake of the organic form of methylmercury [35]. They vary based on the frequency and type of fish consumed [36]. A cross-sectional study reported that higher intake of large predatory fish species was associated with a significantly higher level of blood mercury level among Japanese children [37]. Fish intake exerts several beneficial health effects; however, higher consumption leads to some contradictory outcomes, as fish is considered to be an important confounder that increases blood mercury levels. Among coastal populations residing in Florida, high rates of seafood consumption resulted in elevated levels of blood mercury [38]. Among the Japanese and Korean populations, mercury concentrations were found to be higher in coastal areas than in inland areas, owing to their higher intake of fish and shell-fish-related foods [39,40]. Similarly, in the United States, increased frequency of fish consumption by New York and San Francisco residents was associated with increased blood mercury concentration [41,42]. Studies on the Korean population also have reported that consumption of higher proportions of fish, shellfish, mackerel, oyster, sushi, and sea mustard (more than once a week) increased blood mercury levels [15,19,41]. A general-population study in the Czech Republic showed results consistent with our study, in that women had higher blood mercury concentrations than did men [43]. From all of the studies noted above, it is clear that dietary patterns and certain environmental conditions influence the mercury level in blood. Thus, further studies are needed to analyze the various other factors that contribute to an increased blood mercury level.

Our study showed that blood mercury was not associated with depression in model 1 (demographic and lifestyle factors), which showed mixed results with respect to the effect of fish and mercury on depression. After adjustment for potential confounders (model 2 (fatty fish) and model 3 (model 2+ total fish)), the blood mercury level was significantly associated with increased risk of depression in Korean women. These results suggest that high fish intake may neutralize the effect of mercury on an increased risk of depression, due to high contents of omega-3 fatty acids having protective effects against psychiatric problems such as depression [11]. In our analysis of the association of depression, we found a significantly increased risk of depression for high mercury exposure with low fish intake, but no associations among subjects in higher fish intake group (tertiles 2 and 3, Table 3). As for gender stratification, we found a significant association of depression with high mercury exposure only among women, but not men. However, the mechanisms of the gender difference remain unclear. Recently, depression has been widely recognized as multifactorial, having environmental, cultural, psychological, and socio-economical factors. We can consider several possible factors for women’s susceptibility to diseases: biological factors such as higher body fat, smaller liver, and other hormonal factors, among others [44]. This may suggest a role of estrogen in the immune response to many physical and psychological illnesses. However, plausible mechanisms by which estrogen modulates the host defense system remain to be elucidated. Another possible explanation for the gender difference in the present study is the relatively smaller sample size of men (*n* = 86) compared with women (*n* = 256). According to the National Health and Nutrition Examination Survey (NHANES) data from 1999–2002, blood mercury levels in females are higher in Asians than in Europeans [45]. According to a cross-sectional study on the Korean general population, the mean concentration of total mercury was 2.92 μg/L, and was significantly higher in males (3.11 μg/L) than in females (2.77 μg/L) [46]. Blood mercury in Korean subjects was 3.7–5 times higher than in Germans (0.58 μg/L), Americans (0.70 μg/L), and Canadians (0.79 μg/L) [47,48,49]. Thus, further investigation is needed to clarify the gender differences in the distribution of and susceptibility to mercury intoxication and depression.

The baseline demographic and lifestyle characteristics of our subjects showed significant relationships with a blood mercury level (Table 1). The highest quintile of blood mercury level was associated with older age, obesity, being married, heavy drinking, and higher economic status. Several studies have reported positive associations between blood mercury and obesity among Korean adults [50,51,52,53]. Similarly, higher socio-economic status was associated with higher mercury levels due to the participants’ ability to buy expensive predatory fish such as sharks, tuna, and billfish that contain higher levels of mercury [54]. In our study, increased consumption of white fish, fatty fish such as mackerel, tuna, croaker, and pollack, shellfish and sea food showed high mercury levels among Koreans. Correspondingly, high mercury concentrations have been reported in deep-sea fish such as sharks, tuna, swordfish, smooth hammerheads, crocodile sharks, and shellfish [55].

No previous studies have been reported on the relationship of mercury with fish intake and risk of depression in humans. According to the Korea Food and Drug Administration (KFDA), the total mercury concentration in fish/shellfish and deep-sea fish should be below 0.5 ppm and 1 ppm, respectively [55]. The Stony Brook Medical Center in New York reported that consumption of high-mercury deep-sea fish such as shark, marlin, swordfish, mackerel, tuna, and tile fish was associated with development of fatigue and depressive symptoms [56]. The results of our stratified analysis showed that the association between blood mercury and depression was predominant at the lowest tertile of fish consumption in females. According to KNHANES 2013–2015, consumption of fish greater than four times per week was associated with lower odds of depression among Korean women [57]. Similarly, the Japan Public Health Center-based Prospective Study (JPHC) showed that moderate fish intake can reduce the risk of major depressive disorder [8]. In a Danish National Birth Cohort Study (DNBC), increased risk of postpartum depression was reported for low consumption of fish and n3 polyunsaturated fatty acids (PUFAs) during pregnancy [58]. Recent consideration of the protective effect of fish with high levels of omega-3 fatty acids on depression suggests an important potential role of neuro-inflammation in depression, which might explain why diets low in omega-3 PUFAs are associated with incidence of depressive symptoms [59]. Among the general population of Finland, moreover, incidence of depressive symptoms was significantly higher among infrequent consumers of fish, especially women [60]. Thus, fish consumption has a dual role, in that it positively reduces depressive mood and negatively increases levels of blood mercury.

This study has some limitations. Given its cross-sectional design, we could not infer causality. In addition, as it was observational, interventional studies are needed to obtain stronger evidence regarding the potential benefits of fish consumption for mental health and reduction of depressive symptoms in different ethnic groups. Second, the fact that we did not perform a clinical interview for psychiatric diagnosis could have resulted in misclassification of mental health problems. Third, there is a possibility of missing data on blood of mercury concentration in the survey analysis, which may have resulted from selection bias. Furthermore, survey errors on the food frequency questionnaire (FFQ) might have occurred due to the study participants’ seasonal or daily dietary patterns. Moreover, we estimated fish intake from an interviewer-administered questionnaire, which would not fully reflect exact consumption of fish. We could not estimate n-3 and n-6 PUFA intake from a dietary questionnaire, although epidemiologic studies have suggested the hypothetical association between omega-3 fatty acids and decreased risk of depression. Despite these limitations, the major strength of the present study is that it provides the first report focusing on the relationship between blood mercury and fish intake and its effect on risk of depression among Koreans. Moreover, this study included a large number of participants, which allowed for greater generalizability and consistency in the use of nationally representative data. Additionally, our study results were adjusted for several demographic factors, lifestyles, dietary factors, and different fish types as potential confounders. This study also faithfully followed and ensured all KHNANES procedures respecting blood mercury level and depression evaluation over a long investigative duration, which helped to ensure the integrity of the mercury-concentration data.

The results of the present study indicated an association between blood mercury level and depression among Koreans adults. Higher level of blood mercury was associated with depression among female participants. Additionally, a significant predominant association was observed between blood mercury and depression among female participants with low fish intake. Future research should address the issue of how geographic variability among fish species and consumption of fish from various water bodies effect differences in blood mercury concentration.

## Figures and Tables

**Table 1:** Demographic and lifestyle characteristics of participants by quintiles of blood mercury, KNHANES (2008–2013).

Characteristics	Quintiles of Blood Mercury ^a^					*p*-Value ^b^	Total Subjects
	Q1	Q2	Q3	Q4	Q5		(*n* = 11,754)
	(*n* = 2350)	(*n* = 2351)	(*n* = 2352)	(*n* = 2350)	(*n* = 2351)		
Mercury (range)	0.34–2.73	2.04–3.89	2.74–5.24	3.62–7.55	5.01–168		
Age (years)	43.7 ± 0.52 ^c^	43.5 ± 0.43	44.3 ± 0.38	45.8 ± 0.36	48.5 ± 0.36	<0.0001	45.2 ± 0.17
Gender							
Male	49.6 ^d^	49.6	49.7	49.6	49.6	0.86	49.6
Female	50.4	50.4	50.3	50.4	50.4		50.4
BMI (kg/m^2^)	23.0 ± 0.08	23.6 ± 0.09	23.6 ± 0.08	24.0 ± 0.08	24.5 ± 0.09	<0.0001	23.7 ± 0.04
Underweight (<18.5)	6.41	5.24	4.55	3.84	1.71	<0.0001	4.35
Normal (18.5–22.9)	48.1	42.7	42.1	36.1	31.4		40.1
Overweight (23–24.9)	20.8	23.9	23.4	25.0	25.7		23.8
Obesity (≥25)	24.7	28.2	30.0	35.2	41.2		31.8
Marital status							
Single	36.1	26.2	20.6	16.0	10.3	<0.0001	21.8
Married	63.9	73.8	79.4	84.0	89.7		78.2
Smoking status							
Never	58.9	55.7	54.4	51.8	52.1	0.02	54.6
Former	17.8	19.1	19.8	21.5	22.1		20.0
Current	23.3	25.2	25.8	26.7	25.8		25.4
Alcohol intake ^e^							
Non-drinker	28.2	22.9	22.5	21.2	19.5	<0.0001	22.9
Once a month or under	32.5	30.6	29.5	27.9	25.1		29.1
More than twice a month	31.2	35.9	35.4	36.2	36.0		34.9
Heavy	8.10	10.6	12.6	14.7	19.4		13.1
Household income (1000 Korean won) ^f^							
Low (~750)	30.0	27.9	25.5	23.1	21.1	<0.0001	25.5
Low-intermediate (750~1500)	26.6	25.7	24.7	24.3	24.1		25.1
Upper-intermediate (1500~2460)	24.0	24.9	25.1	25.7	23.5		24.6
High (2460~)	19.4	21.5	24.7	26.9	31.3		24.8
Physical activity (MET-h/d)	35.6 ± 62.0	41.1 ± 62.3	40.7 ± 66.4	44.4 ± 70.1	45.2 ± 68.9	0.03	41.1 ± 66.1
Total energy intake (kcal/day)	1943 ± 23.7	2005 ± 24.3	2017 ± 23.5	2012 ± 25.1	2026 ± 24.4	0.09	2000 ± 10.6
Total fish intake (freq/wk) ^g^	4.37 ± 0.13	5.28 ± 0.13	5.91 ± 0.12	6.05 ± 0.13	6.57 ± 0.15	<0.0001	5.58 ± 0.06
Lowest quintile1	30.4	22.6	17.3	15.8	14.5	<0.0001	20.0
Quintile2	22.5	21.0	20.6	18.6	17.9		20.1
Quintile3	18.0	18.7	21.4	21.7	19.6		19.9
Quintile4	16.2	20.1	19.9	21.4	22.0		20.0
Highest quintile5	12.9	17.6	20.8	22.5	26.0		20.0
White fish (freq/wk)	0.51 ± 0.02	0.67 ± 0.03	0.79 ± 0.03	0.87 ± 0.03	1.04 ± 0.04	<0.0001	0.76 ± 0.01
Fatty fish (freq/wk)	2.16 ± 0.07	2.44 ± 0.07	2.82 ± 0.08	2.75 ± 0.07	2.99 ± 0.08	<0.0001	2.61 ± 0.03
Other fish (freq/wk)	0.96 ± 0.04	1.05 ± 0.04	1.12 ± 0.04	1.05 ± 0.03	1.01 ± 0.04	0.06	1.04 ± 0.02
Shellfish (freq/wk)	1.32 ± 0.06	1.66 ± 0.07	1.60 ± 0.06	1.75 ± 0.06	1.97 ± 0.07	<0.0001	1.58 ± 1.91

BMI, body mass index; MET, metabolic equivalent task. ^a^ Blood mercury is divided into five groups. Q1 and Q5 are the lowest and highest quintile groups, respectively. ^b^ ANOVA and chi-square tests are used to assess the significance for continuous and categorical variables, respectively. ^c^ Continuous values are presented as mean and standard error. ^d^ Categorical values are presented as %. ^e^ Alcohol intake was categorized into four groups, including non-drinker, ≤1 time/month, ≥2 times/month, and heavy drinker. ^f^ Household income was calculated by dividing the monthly income by the number of family members. ^g^ White fish (croaker, pollack, hairtail), fatty fish (mackerel, tuna, anchovy), other fish (fish cake, squid, salted seafood) and shellfish (clam).

**Table 2:** Multivariate logistic regression for association between depression and blood mercury for all participants, KNHANES (2008–2013).

Variables	Quintiles of Blood Mercury ^a^					*p*-Trend ^b^	Continuous of Blood Mercury
	Q1	Q2	Q3	Q4	Q5		
Total (*n* = 11754)	2350	2351	2352	2350	2351		
Blood mercury (Mean ± SE)	1.79 ± 0.01 ^c^	2.83 ± 0.01	3.82 ± 0.02	5.22 ± 0.03	9.80 ± 0.18		
Depression cases (n = 342)	63 (2.68) ^d^	60 (2.55)	66 (2.81)	75 (3.20)	78 (3.32)	0.163	
Multivariate Model 1 ^e^	Ref	1.09 (0.73–1.63)	1.05 (0.66–1.66)	1.18 (0.76–1.82)	1.41 (0.91–2.18)	0.167	1.03 (1.02–1.05)
Multivariate Model 2 ^f^	Ref	1.32 (0.81–2.16)	1.32 (0.83–2.10)	1.40 (0.89–2.21)	1.77 (1.12–2.78)	0.027	1.03 (1.02–1.05)
Multivariate Model 3 ^g^	Ref	1.31 (0.80–2.15)	1.33 (0.84–2.11)	1.39 (0.88–2.20)	1.76 (1.12–2.76)	0.03	1.03 (1.02–1.05)
Male (*n* = 5834)	1166	1167	1168	1166	1167		
Blood mercury (Mean ± SE)	2.01 ± 0.02	3.29 ± 0.01	4.53 ± 0.01	6.24 ± 0.02	12.1 ± 0.30		
Depression cases (n = 86)	18 (1.55)	14 (1.20)	21 (1.80)	19 (1.64)	14 (1.20)	0.584	
Multivariate Model 1	Ref	0.80 (0.32–2.02)	1.52 (0.66–3.54)	1.13 (0.48–2.63)	0.94 (0.39–2.26)	0.794	1.03 (1.02–1.05)
Multivariate Model 2	Ref	0.72 (0.26–2.00)	1.66 (0.71–3.86)	1.24 (0.52–2.98)	0.95 (0.37–2.44)	0.624	1.03 (1.02–1.05)
Multivariate Model 3	Ref	0.72 (0.26–1.98)	1.65 (0.71–3.86)	1.23 (0.51–2.97)	0.95 (0.38–2.40)	0.629	1.03 (1.02–1.05)
Female (*n* = 5920)	1184	1184	1184	1184	1184		
Blood mercury (Mean ± SE)	1.56 ± 0.01	2.38 ± 0.01	3.15 ± 0.01	4.23 ± 0.01	7.57 ± 0.11		
Depression cases (n = 256)	45 (3.80)	46 (3.89)	45 (3.81)	56 (4.73)	64 (5.41)	0.211	
Multivariate Model 1	Ref	1.19 (0.76–1.86)	0.93 (0.53–1.61)	1.20 (0.72–1.99)	1.54 (0.93–2.56)	0.168	1.03 (0.97–1.10)
Multivariate Model 2	Ref	1.56 (0.89–2.75)	1.24 (0.71–2.16)	1.49 (0.88–2.52)	2.07 (1.22–3.51)	0.031	1.04 (1.00–1.09)
Multivariate Model 3	Ref	1.55 (0.88–2.73)	1.24 (0.71–2.17)	1.47 (0.87–2.49)	2.05 (1.20–3.48)	0.036	1.04 (1.00–1.09)

^a^ Blood mercury was divided into five groups by quintile. Q1 and Q5 are the lowest and highest quintile groups, respectively. ^b^ *p*-values are calculated for the linear trends of multivariate odds ratios (OR). ^c^ Values are presented as mean and standard deviation. ^d^ Values are presented as N (%). ^e^ Adjusted for age (years), physical activity (MET-h/day) and energy intake (kcal/day) as continuous variables and for gender (male, female), smoking status (non-smoker, past-smoker, smoker), drinking status (non-drinker, ≤1 time/month, ≥2 times/month, heavy drinker), and household income (low, low-intermediate, upper-intermediate, high) as categorical variables in addition to covariates in age-adjusted model. ^f^ Adjusted for fatty fish intake (freq/week) in addition to covariates in multivariate model 1. ^g^ Adjusted for total fish intake (freq/week) in addition to covariates in multivariate model 1.

**Table 3:** Multivariate logistic regression for association between depression and blood mercury, by gender and fish intake (KNHANES 2008–2013).

Variables	Quintiles of Blood Mercury ^a^					*p*-Trend ^b^	Continuous of Blood Mercury
	Q1	Q2	Q3	Q4	Q5		
Male (*n* = 5834)	1166	1167	1168	1166	1167		
Depression cases (*n* = 86)	18 (1.55) ^c^	14 (1.20)	21 (1.80)	19 (1.64)	14 (1.20)	0.584	
Total fish tertile 1 (0.00–2.97, *n* = 1482) ^d^							
Age adjusted ^e^	Ref	0.26 (0.06–1.12)	0.46 (0.14–1.54)	0.32 (0.09–1.11)	0.26 (0.05–1.25)	0.059	0.81 (0.65–1.02)
Multivariate adjusted ^f^	Ref	0.35 (0.08–1.55)	0.74 (0.21–2.59)	0.53 (0.14–2.05)	0.51 (0.11–2.47)	0.356	0.91 (0.76–1.08)
Total fish tertile 2 (2.98–6.64, *n* = 1479)							
Age adjusted	Ref	3.72 (0.36–38.5)	6.75 (0.67–68.1)	7.39 (0.84–64.9)	2.12 (0.21–21.9)	0.364	0.98 (0.92–1.04)
Multivariate adjusted	Ref	3.86 (0.37–40.9)	7.64 (0.77–75.4)	8.10 (0.90–72.7)	2.53 (0.23–27.8)	0.268	1.01 (0.93–1.05)
Total fish tertile 3 (6.65–76.2, *n* = 1480)							
Age adjusted	Ref	1.17 (0.15–9.03)	3.57 (0.65–19.7)	0.95 (0.15–6.18)	2.25 (0.39–13.1)	0.526	1.04 (1.02–1.05)
Multivariate adjusted	Ref	1.28 (0.15–10.8)	4.10 (0.71–23.6)	1.19 (0.18–7.81)	2.91 (0.45–18.9)	0.325	1.04 (1.03–1.06)
Female (*n* = 5920)	1184	1184	1184	1184	1184		
Depression cases (*n* = 256)	45 (3.80)	46 (3.89)	45 (3.81)	56 (4.73)	64 (5.41)	0.211	
Total fish tertile 1 (0.00–3.03, *n* = 1703)							
Age adjusted	Ref	2.94 (1.14–7.60)	1.78 (0.69–4.55)	2.74 (1.07–7.01)	3.39 (1.25–9.15)	0.042	1.06 (0.98–1.15)
Multivariate adjusted	Ref	3.15 (1.21–8.22)	1.90 (0.74–4.86)	2.92 (1.13–7.57)	4.00 (1.51–10.6)	0.015	1.07 (1.00–1.15)
Total fish tertile 2 (3.04–6.62, *n* = 1696)							
Age adjusted	Ref	0.85 (0.33–2.21)	0.53 (0.19–1.51)	0.87 (0.36–2.14)	1.07 (0.45–2.53)	0.851	0.96 (0.87–1.06)
Multivariate adjusted	Ref	0.95 (0.38–2.41)	0.68 (0.25–1.87)	0.98 (0.40–2.43)	1.37 (0.56–3.34)	0.553	0.99 (0.90–1.09)
Total fish tertile 3 (6.63–43.2, *n* = 1702)							
Age adjusted	Ref	1.14 (0.35–3.74)	1.20 (0.41–3.50)	1.00 (0.36–2.80)	1.40 (0.51–3.86)	0.640	1.04 (0.95–1.13)
Multivariate adjusted	Ref	1.07 (0.33–3.48)	1.25 (0.44–3.56)	1.08 (0.39–3.02)	1.55 (0.56–4.31)	0.422	1.05 (0.97–1.14)

^a^ Blood mercury is divided into five groups by quintile. Q1 and Q5 are the lowest and highest quintile groups, respectively. ^b^ *P* values are calculated for the linear trend of multiple odds ratios (OR). ^c^ Values are presented as N (%). ^d^ Total fish intake are divided into three groups by gender. T1 and T3 are the lowest and highest tertile groups, respectively. ^e^ Adjusted for age (years) as continuous variable. ^f^ Adjusted for physical activity (MET-h/day) and energy intake (kcal/day) as continuous variables and for smoking status (non-smoker, past-smoker, smoker), drinking status (non-drinker, ≤1 time/month, ≥2 times/month, heavy drinker), and household income (low, low- intermediate, upper-intermediate, high) as categorical variables.
